# Fundamentals of T Cell Metabolism and Strategies to Enhance Cancer Immunotherapy

**DOI:** 10.3389/fimmu.2021.645242

**Published:** 2021-03-18

**Authors:** Guillermo O. Rangel Rivera, Hannah M. Knochelmann, Connor J. Dwyer, Aubrey S. Smith, Megan M. Wyatt, Amalia M. Rivera-Reyes, Jessica E. Thaxton, Chrystal M. Paulos

**Affiliations:** ^1^Department of Microbiology and Immunology, Medical University of South Carolina, Charleston, SC, United States; ^2^Department of Surgery, Emory University, Atlanta, GA, United States; ^3^Department of Microbiology and Immunology, Emory University, Atlanta, GA, United States; ^4^Department of Orthopaedics and Physical Medicine, Medical University of South Carolina, Charleston, SC, United States

**Keywords:** T cell metabolism, tumor microenvironment, adoptive T cell transfer, immune checkpoint therapy, tumor metabolism, tumor infiltrating lymphocytes

## Abstract

Emerging reports show that metabolic pathways can be targeted to enhance T cell-mediated immunity to tumors. Yet, tumors consume key metabolites in the host to survive, thus robbing T cells of these nutrients to function and thrive. T cells are often deprived of basic building blocks for energy in the tumor, including glucose and amino acids needed to proliferate or produce cytotoxic molecules against tumors. Immunosuppressive molecules in the host further compromise the lytic capacity of T cells. Moreover, checkpoint receptors inhibit T cell responses by impairing their bioenergetic potential within tumors. In this review, we discuss the fundamental metabolic pathways involved in T cell activation, differentiation and response against tumors. We then address ways to target metabolic pathways to improve the next generation of immunotherapies for cancer patients.

## Introduction

It has long been appreciated that glycolysis and mitochondrial respiration work together to satisfy the long-term energetic demands of T cells in the host (1). As T cell survival is often impaired in patients with cancer and chronic infectious disease (1, 2), it is necessary to have an effective metabolic capacity for a productive immune response (1). For example, in patients, one reason T cells do not thrive amidst tumor cells is that they compete for the same energy sources (1). Herein, we review the fundamental metabolic requirements for T cells to survive, proliferate and mount antigen-specific responses in the context of effector and memory responses. We then outline how the harsh tumor microenvironment manipulates T cell metabolism to impair effector functions. Finally, we contemplate emerging data where metabolic manipulations have been performed and have shown promise for augmenting T cell-based immunotherapies for patients with cancer.

## A Brief History of T Cell Metabolism

Studies in the mid-20^th^ century first detailed the nutrient requirements for quiescent and activated T cells to survive. It was discovered that energy production and nutrient uptake shifts when a resting T cell is activated *via* signaling cues (3, 4). In the 1960’s, work by Hedeskov et al. initially described the metabolism of T lymphocytes at the resting state. Surprisingly, resting T cells largely depended on oxidative phosphorylation (OXPHOS) to survive. Additional investigations, published nearly a decade later, uncovered that resting T cells shift from OXPHOS to avid glycolysis and amino acid consumption upon TCR-mediated recognition of antigen (5). While this finding is obvious now, it was unexpected at the time, especially given that exploiting glycolysis for energy was largely thought less efficient than OXPHOS for T cells to generate ATP (3). For many years, these observations remained as descriptive findings of the highly dynamic ways T cells use bioenergetics to thrive. However, from the 1980’s to present day, the significance of bioenergetic requirements for the activation, effector functions and lasting memory of T cell responses against tumors have begun to be elucidated and exploited to improve medicine.

**Figure 1:**
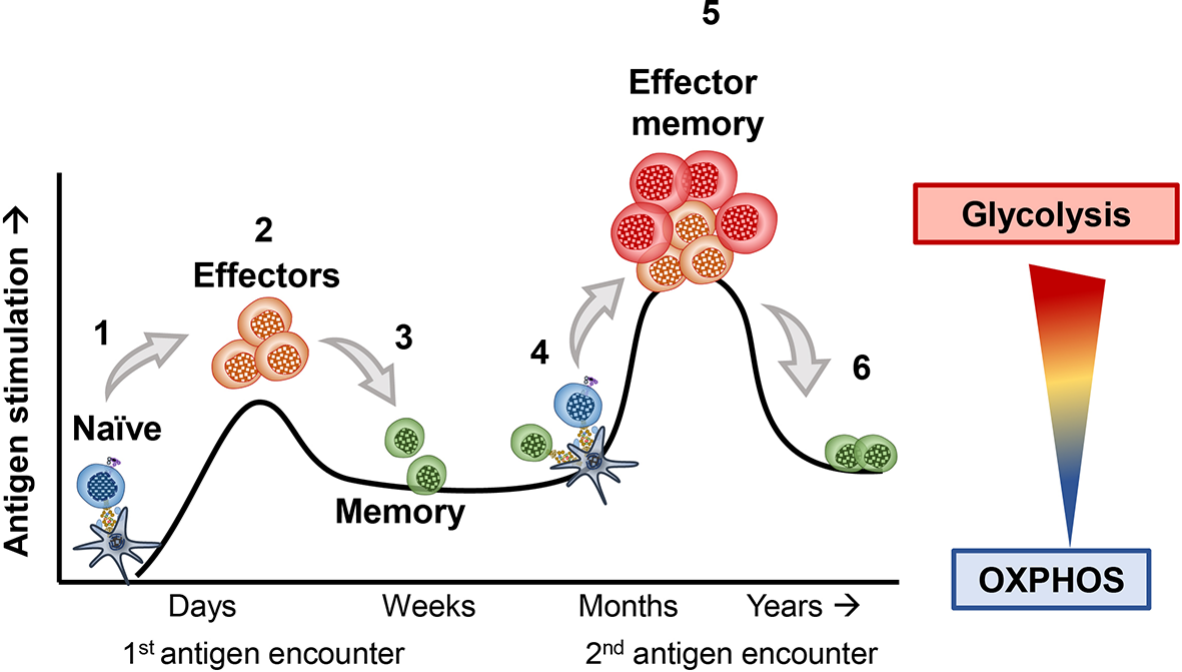
1) Naive T cells breakdown glucose and efficiently break it down through the tricarboxylic acid (TCA) cycle and oxidative phosphorylation (OXPHOS) to survive, until they encounter their antigen. 2) Upon a primary exposure to antigen, naïve T cells differentiate into effector T cells. As effectors they shift towards the use of amino acids as well as glucose, both required for their proliferation and cytolytic activity. 3) After clearing their inciting antigen, many effector T cells die. However, a fraction of surviving T cells can form memory T cells, which adapt towards improved mitochondrial biogenesis and OXPHOS. 4) These memory T cells can survive for many months to years until they encounter a similar antigen. 5) If these memory T cells re-encounter the same antigen, they rapidly become effectors and more efficiently engage in glycolysis and amino acid usage to robustly proliferate and secrete cytokines. 6) The T cells that survive maintain their usage of OXPHOS to persist long-term within hosts.

T cells use different metabolic pathways based on their differentiation and memory status (6–8). **Figure 1** visually portrays how T cells exploit distinct metabolic pathways throughout their lifetime and during encounters with foreign antigen, such as viruses or transformed cells (9–11). As mentioned, naïve T cells rely on OXPHOS to survive in their resting state (12, 13). However, upon primary exposure to antigen, naïve T cells differentiate into effector cells and use glycolysis to help them effectively secrete cytokines, such as IFN-gamma and TNF-alpha (14–17). Following activation, naïve T cells shift from mostly oxidizing glutamine to lactate (75% of lactate produced from glutamine oxidation) through OXPHOS towards mostly using anaerobic glycolysis and partial glutamine oxidation (67% of all lactate from glucose metabolism, and 33% from glutamine), surprisingly without significantly changing their ATP production (5). After effector T cells encounter an antigen challenge, many of them die (18). However, a few prevail and survive long-term to battle re-infections or tumor relapse (17, 19, 20). These T cells are termed memory T cells. When memory T cells encounter the same antigen, they can more rapidly induce their effector functions to clear the insult (6, 21). These T cells are termed effector memory cells (EM) (22, 23). Effector T cells derived from memory rather than antigen naïve precursors more efficiently produce cytolytic cytokines by improving the coupling of glycolytic enzymes and mitochondrial machinery to rapidly utilize glucose following a secondary encounter with antigen (11, 24). Most effector memory T cells perish, but the few survivors employ OXPHOS to persist (8, 25). Below, we elaborate on the metabolic requirements of T cells at various stages of differentiation.

## Metabolic Requirements Distinguish Effector and Memory T Cell Subsets

### Naïve T Cell Metabolism

**Figure 2:**
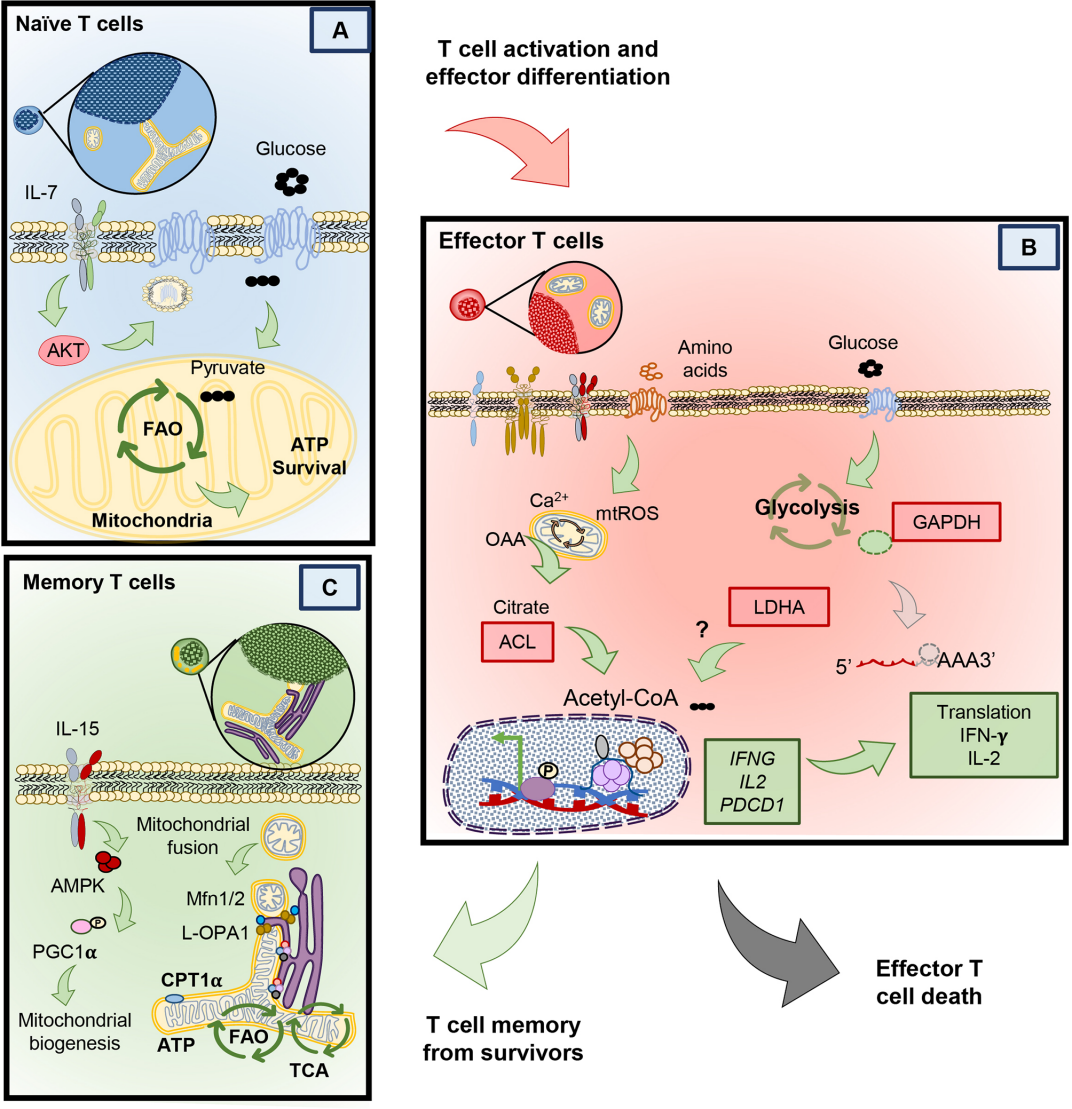
Different metabolic programs between naïve, memory and effector T cells. **(A)** Naïve T cells rely on the full oxidation of glucose through OXPHOS, in the absence of TCR stimulation. **(B)** Upon T cell stimulation T cells undergo protein and transcriptional changes in metabolism that allow the sustained activity of glycolysis and other amino acid uptake and usage. Glycolysis by products in effector T cells mediate changes that help sustain effector cytokine release and cytolytic function. Effector T cells that clear antigen either die or contract to form memory T cells. **(C)** Compared to effectors, memory T cells possess an enhanced metabolic profile dependent on mitochondrial biogenesis, mitochondrial fusion and reliance on fatty acid oxidation.

Naïve T cells can live for the entire duration of the host’s life. In fact, naïve T cells can be detected in humans as old as 100 years (26). Only after they encounter their respective antigens, do they either become effector T cells that perish or transition into memory T cells that continue to thrive (19). But how do naïve cells remain viable for so long? As in **Figure 2A**, naïve T cells can only survive when homeostatic cytokines, like interleukin 7 (IL-7) provide signaling cues (27). IL-7 provides the signaling necessary to enable the mechanisms that nurture the survival of naïve T through Akt signaling (28). This pathway, in naïve T cells promotes the translocation of the glucose transporter 1 (Glut1) to take up glucose. Glucose is then broken down into pyruvate, a substrate that enters the mitochondria to activate the synthesis of triacyl glycerol, which serves as a source of lipids that fuels into the fatty acid oxidation (FAO) pathway (28–30). In contrast to this maintenance phase, many nutrients (glucose, glutamine, L-arginine, and other amino acids) are needed to differentiate naïve T cells into the effector phenotype upon antigen encounter (5). We next will detail how activated T cells engage in transcriptional and metabolic changes to license them to proliferate and secrete effector cytokines.

### Glycolysis in Effector T Cell Function

Naïve T cells become activated upon TCR engagement with an antigen presented *via* the major histocompatibility complex (MHC) on antigen presenting cells (signal 1). However naïve T cells require a second signal *via* costimulatory molecules (signal 2) to become fully activated and proliferate (31–33). Along with these two signals, cytokines in the host play a key role in fine tuning the fate and metabolic profile of naïve T cells into either an effector or memory phenotype (**Figure 2B**) (12, 34–36). Effector T cells secrete cytotoxic cytokines, such as interferon-γ, TNF-α and granzyme B. These cytokines and cytotoxic molecules destroy cancer cells or clear viruses (37–39). T cells require many metabolic resources to mediate clearance of these foreign antigens. However, instead of engaging in the highly energetically favorable OXPHOS pathway, effector T cells use Warburg metabolism to proliferate and to produce cytokines (24, 40, 41). Warburg metabolism, initially discovered as an important pathway for the survival of malignant cells, is characterized by an extraordinary ability to breakdown glucose by anaerobic glycolysis and amino acids such as glutamine (as in **Figure 2B**) (42, 43). In contrast to naïve T cells, effectors break down glucose to pyruvate and lactate with minimal engagement of mitochondrial respiration (44–46).

Although the metabolic adaptations T cells undergo when activated may appear obvious given the increased energetic demand to proliferate and synthesize proteins, recent findings suggest that these changes are tightly coupled to T cell differentiation and acquisition of effector function. Interestingly, two key enzymes in the anaerobic glycolysis pathway— GAPDH and LDHA—are critical in regulating cytokine production in T cells. Glyceraldehyde 3-phosphate Dehydrogenase (GAPDH), aside from its role in metabolizing glucose, can directly bind the mRNA of key cytokines such as *IL-2*, *IFNG* and *TNFA* in CD4^+^ effector T cells to prevent their protein translation in the absence of glucose **(**right side of **Figure 2B**) (41). In contrast to preventing the direct protein translation of cytokines by GAPDH in CD4^+^ T cells, CD8^+^ T cells instead utilize lactate dehydrogenase (LDHA), the key enzyme in the conversion of pyruvate into lactate for anaerobic glycolysis, to enforce effector gene expression *via* histone acetylation (46, 47). Genetic loss of *LDHA* prevents acetylation at the promoters of effector genes such as *IFNG* and *PDCD1*, without compromising proliferation (41, 48). Although glucose is a critical metabolite for T cell function, the enzymes involved in anaerobic glycolysis are also tightly coupled to effector function in both CD4^+^ and CD8^+^ T cells (49). A potential mechanism explaining the functional effect of using glycolysis to promote effector functions may lie in production of citrate downstream of glucose breakdown. Citrate is shuttled from the mitochondria where it is converted into acetyl-coA by the action of cytosolic ATP Citrate Lyase (ACL) (50). ACL is an enzyme that is upregulated in both CD4^+^ and CD8^+^ effector T cells that can translocate from the cytoplasm to the nucleus and has the ability to directly acetylate histones of effector gene promoters (49). Thus, glycolysis regulates effector T cell functionality; while CD4^+^ T cells moonlight GAPDH to regulate cytokine translation, both CD4^+^ and CD8^+^ T cells use acetyl co-A to regulate histone acetylation of effector genes **(**left side of **Figure 2B**).

In addition to glucose, amino acids are critical for T cell proliferation and function. For example, glutamine breakdown in T cells is required for their proliferation but not for their cytokine production (48, 51). In fact, in the absence of L-Glutamine, T cells are unable to proliferate but can still secrete cytokines (48, 52). These findings suggest synergism from the breakdown of glucose and amino acids (such as glutamine) for T cell proliferation and effector functions. How other amino acids regulate T cell function under nutritional stress remains poorly understood, but is likely to be essential for immunity to tumors as we will discuss in later sections.

Because malignant cells use the same nutrients as effector T cells, they compete form them to thrive. Deprivation of glucose or glutamine in the tumor microenvironment vastly impairs T cell proliferation, function and survival (53–57). Although there may be recent findings that suggest inhibiting glutamine metabolism in the tumor may benefit T cells while impairing tumor metabolism (58). Often this tug of war forces effector T cells to use alternative carbon sources to survive (59–61). It is now clear that T cells use glycolysis to sustain their inflammatory potential, not only as a means to an end, but also as a regulatory component in T cell immunity.

Although metabolic changes permit effector T cells to become highly inflammatory, they come at the price of compromising their mitochondrial quality and capacity to self-renew (36, 62). However, the small T cell fraction that survive the initial antigen encounter acquire a different set of metabolic adaptations to prevail longer-term (63). Next, we discuss how changes to mitochondrial metabolism and morphology impact the development of memory T cells and their recall capacity.

### Mitochondrial Properties of Effector and Memory T Cells

Memory T cells develop after a primary antigen challenge, persisting from the pool of lymphocytes with specific metabolic adaptations permitting self-renewal and survival long-term (**Figure 2C**) (64). Given the vast differences in function comparing effector and memory cells, alteration of mitochondrial networking and morphology is critical to fulfill the metabolic needs of these T cells. For example, mitochondria are recruited to the immune synapse after an antigen encounter in effector cells following cleavage from mitochondrial-endoplasmic reticulum (ER) contact sites to enable calcium influx and T cell activation (**Figure 2B**) (65). In contrast, as memory T cells develop they re-organize their mitochondria to associate tightly with the ER, a feature lacking in terminal effector and naïve T cells (66), which provides a pool of mitochondria primed to sustain aerobic glucose metabolism (67), directly enhancing IFN-gamma production during a secondary response to antigen.

Further, remodeling of mitochondrial morphology is critical for the specialized metabolic needs of effector versus memory T cells. In effector cells, mitochondrial fragmentation, also called fission, produces mitochondria with loose cristae and poorly efficient electron transport but high capacity to buffer calcium (68–71). This morphological and functional change enables the production of reactive oxygen species (ROS) and upregulation of anaerobic glycolysis, needed for the expression of NFAT, a transcription factor required for T cell activation (70, 72–74). In contrast to effectors, memory T cells adapt their mitochondrial morphology for cell-intrinsic usage of lipids and FAO (25). Memory T cells undergo mitochondrial fusion to protect against DNA damage from accumulated ROS to sustain survival under nutritional restriction (71, 75). Cells that acquire the tubular network of fused mitochondria produce less ROS, have tight cristae arrangement and electron transport complexes in close proximity to each other, indicating efficient mitochondrial respiration (70, 76). For example, spare respiratory capacity (SRC) and ATP production is elevated in memory T cells, indicating that they shift towards OXPHOS metabolism with reduced mtROS (36).

Due to these robust differences in metabolic state, manipulation of mitochondrial properties is an active area of research to direct T cells to specific phenotypes. Mitochondrial respiration can be driven by many different types of fuel. For example, IL-7 and IL-15 support the survival of memory T cells, in part, by inducing mitochondrial biogenesis and allowing utilization of alternative substrates to glucose for FAO, such as long chain fatty acids and triacylglycerols (**Figure 2C**) (11, 35). Seminal work by the Pearce group and others demonstrated that spare respiratory capacity and FAO was key for the development of T cell memory (8, 11, 36). Importantly, memory T cell formation could be induced by AMP-dependent-Protein Kinase (AMPK) activity *via* metformin, an FDA approved drug for diabetes (8).

AMPK is a serine threonine kinase responsive to AMP production or energy depletion and has critical function in the development of memory T cells without compromising a primary antigen challenge (77, 78). Mitochondrial respiration and memory formation are compromised in T cells deficient in the catalytic subunit of AMPK (63). In fact, AMPK is a critical regulator of the mitochondrial biogenesis transcription factor, peroxisome proliferator-activated coactivator 1α (PGC1α), which bolsters mitochondrial formation (**Figure 2C**) (79, 80). The importance of mitochondrial biogenesis and function has been recently highlighted by studies showing that either induction of PGC1α through 4-1BB signaling or genetic its overexpression in T cells enhances memory formation against tumors (81, 82). Based on our understanding of how metabolism and mitochondrial homeostasis changes through a T cell’s lifetime, under nutrient competent environments, we next discuss how T cell metabolism is altered in the tumor.

## Nutritional Tug of War: T Cells vs the Tumor Microenvironment

### Nutrient Competition

**Figure 3:**
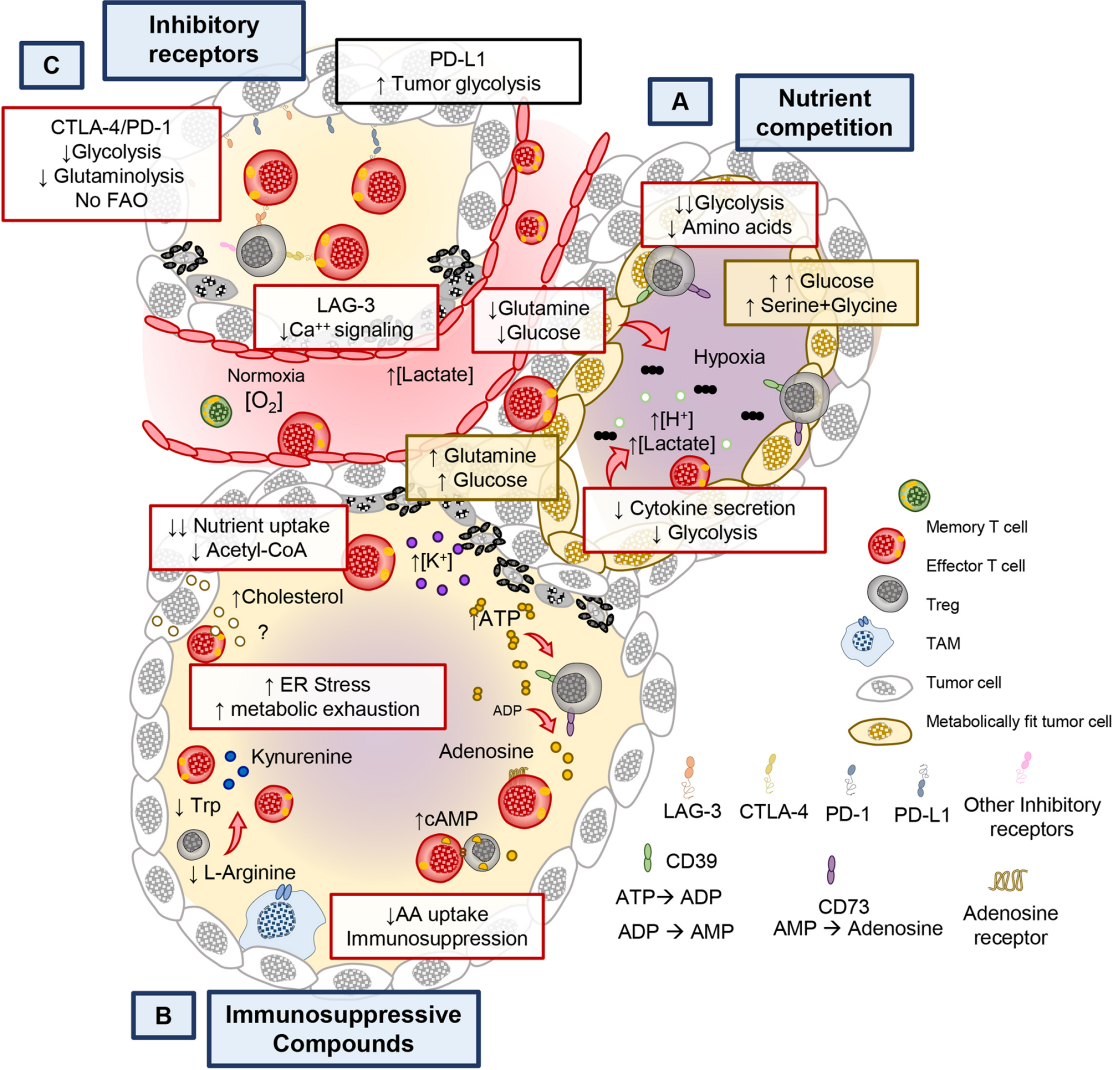
Metabolic and immunological checkpoints that hinder T cell mediated tumor immunity. **(A)** Tumors can adapt their metabolism in response to nutritional stress to better compete and scavenge for glucose and amino acids to suppress T cell bioenergetics. **(B)** Chronic stimulation in the tumor bed leads to the expression of immune checkpoint receptors such as PD-1/PD-L1, CTLA-4, LAG-3, and they exert negative metabolic functions in T cells. **(C)** Furthermore, Ionic imbalances, oxygen availability, and metabolites impact the function of T cells. By products of immunosuppressive immune cells, cell debris and tumor metabolites create the conditions that contribute to the metabolic exhaustion of tumor specific T cells.

It has long been appreciated that the cytotoxic potential of CD8^+^ (CTL) T cells is impaired in the tumor (83). Emerging reports reveal that tumors and activated T cells share common metabolic programs to survive, thus setting the stage for a continuous battle (or tug of war) for nutrients (40, 42, 84). Several lines of evidence support this notion as tumors with gain-of-function mutations in enzymes involved in glycolysis have increased resistance to T cell mediated immunity. This feature presides independent of checkpoint inhibitory receptor expression (84). For example, in renal cell carcinoma, Glut1 expression in tumors is inversely correlated to CD8^+^ T cell infiltration and cytolytic capacity (43). Moreover, solid tumors are composed of heterogenous populations with differing metabolic adaptations that outcompete T cells in consuming glutamine, glucose and amino acids (**Figure 3A**). Within hypoxic regions, tumors use glucose and glutamine *via* the action of HIF-1α, a hypoxia inducible transcription factor, critical for maintaining glucose and glutamine breakdown under oxygen stress (84, 85). The same mechanism that allows tumors to thrive can further hinder the anti-tumor potential of T cells as hypoxia sensed by prolyl-hydroxylase (PHD) proteins can prevent T cell protection against metastatic lesions in the lungs by downregulating glycolysis genes (86). Because of the heterogenous nature of the tumor mass, areas of hypoxia allow for the development of highly glycolytic tumor regions that contribute to the acidic tumor microenvironment (TME) (87, 88). This contribution can be attributed, in part, to lactate secretion, which relies in proton co-transporters and can be detrimental to T cell activation (89, 90). Lactate must be exported out of the cell along with H^+^ ions to maintain homeostasis and to sustain glycolysis (91). When exported by tumor cells, lactate hinders T cell activation by altering the gradient across lactate transporters, thereby preventing recycling of glycolytic byproducts and preventing glycolysis in T and NK cells (87–89, 92). Lactate and proton build up leads to acidification (pH <6.4) of the tumor, in turn blunting T cell effector functions (93, 94). Furthermore, recent evidence suggests that lactate can serve as a substrate to promote immunosuppressive populations of regulatory T cells (T_regs_) present in the TME (95, 96).

### Metabolism of Intratumoral Tregs

Regulatory T cells (T_regs_) can directly and indirectly blunt cytotoxic CD8^+^ T cell response against the tumors (97). Similarly to conventional inflammatory CD4 T cells, T_regs_ can induce the glycolytic machinery upon TCR engagement, however T_regs_ complement their metabolism by inducing fatty acid biosynthesis and oxidative phosphorylation which allows them to survive longer than their inflammatory counterparts (98). In fact, T_regs_ rely on the expression and function of the electron transport chain complex III to sustain their suppressive function, as deletion of components of complex III leads to fatal autoimmunity within 25 days in mice and promote tumor immunity in B16 melanoma tumors after inducible deletion (99). The reliance on fatty acid metabolism and the respiratory chain provides T_regs_ with a metabolic advantage to thrive within tumors as they have scarce levels of glucose available and produce high lactate levels, a metabolic state that not only blunts cytotoxic activity but also provides an alternative fuel source to tumor infiltrating T_regs_ (100). As noted with deletion of complex III, a targetable vulnerability of tumor infiltrating T_regs_ exists and can be exploited to destabilize their suppressive function. Indeed, T_regs_ stability can be perturbed when CTA-4 blockade is used in glycolysis impaired tumors, through metabolic reprograming of T_regs_ towards glycolysis and a skewing towards an inflammatory phenotype, a process that is inhibited when tumors have high glycolytic capacity (101). This finding has tremendous potential for translation into the clinic, as it can be targeted using pharmaceutical agents.

### Immunosuppressive Compounds

Tumors indirectly deprive effector CD8^+^ and helper CD4^+^ T cells of the metabolic nutrients required for their function and survival. One example of this scarcity of nutrients for immune cells is driven by the accumulation of potassium [K+] in the interstitial fluid of the tumor, which acts to suppress transporters for amino acid and glucose in T cells (**Figure 3B**) (102). Nutrient deprivation depletes the nucleocytosolic pools of acetyl CoA in T cells, preventing the acetylation of the *IFNG* promoter and therefore impairing their production of IFNγ (49, 103). This pathway plays a key role in modulating the epigenetic landscape of effector T cells.

Another mechanism of indirect nutrient deprivation is mediated by the byproducts of suppressive T_reg_ cells, tumor cells, and other suppressive immune cells within the TME. T_regs_ produce adenosine in tumors by CD39/CD73-mediated catalysis (ATP → ADP → Adenosine, as shown in **Figure 3B**. Adenosine is a suppressive molecule that binds to adenosine receptors (A2AR) on cytotoxic T cells and suppresses their function *via* reducing NfkB signaling (104) or by inducing suppressive function on regulatory T cells (105). Furthermore, tumor metabolic byproducts, such as cholesterol, can induce metabolic stress in T cells. Specifically, tumor derived cholesterol induces ER stress which prevents the ability of T cells to secrete cytokines. Furthermore, the ER stress response promotes the factor XBP-1 which can directly increase PD-1, TIM-3, and LAG-3 expression, important immunosuppressive molecules that mediate T cell exhaustion (106). Not only do tumors secrete immunosuppressive molecules, but also other immune cells take up nutrients that are beneficial to T cells and can produce immunosuppressive metabolites. For example, M2 type macrophages in the tumor consume L-arginine in an arginase-1 dependent manner and can deplete tryptophan by breaking it down into immunosuppressive kynurenine derivatives through indoleamine-2,3-oxygenase (IDO) (56). These are just a few of the mechanisms that drive metabolic cross talk between tumors and immune cells. Thus, many byproducts of cellular metabolism synergize in the tumor to suppress T cells from fulfilling their potential to eradicate tumors and are likely to also play an obstacle in the growth of TILs from tumor biopsies (**Figure 3B**). In addition to this metabolic tug of war, the effector functions of T cells are limited by inhibitory receptor on tumors and immunosuppressive host elements, such as myeloid and T_regs_ cells.

## Immune Checkpoints in T Cell Metabolism

Tumors evade the immune system in order to survive in the host. Tumors do this in many ways, as depicted in **Figure 3C**. One such mechanism is by promoting T cell exhaustion (107). T cells that become exhausted had a reduced capacity to survive, proliferate and secrete cytokines (108). T cell dysfunction is marked by the progressive acquisition of inhibitory receptors (IRs), including programmed cell death protein 1 (PD-1), lymphocyte activation gene-3 (LAG-3), cytotoxic T-lymphocyte association protein 4 (CTLA4), and T cell immunoglobulin and mucin domain-containing protein 3 (TIM3) (109–111). These IRs alter T cell responses against tumors in part by perturbing their metabolism.

T cell function and proliferation are compromised *via* immune checkpoint inhibitory pathways in the tumor (15, 112). PD-1 impairs effector function by downregulating glycolysis and increasing the FAO rate limiting enzyme CPT1α, a feature that supports T cell persistence in the tumor but prevents their cytotoxic potential (112). Although mitochondrial FAO supports T cell persistence but not function, PD-1^+^ T cells exhibit markedly decreased mitochondrial respiration (**Figure 3C**) (112, 113). Furthermore, Akt signaling is elevated in tumor infiltrating lymphocytes experience, which potent inhibits PGC1α, a key regulator of mitochondrial biogenesis (81, 113). This data suggests that part of the suppressive mechanism of PD-1 and chronic antigen stimulation is attributed to their negative effect on T cell mitochondrial biogenesis, substrate utilization and glycolytic capacity (**Figure 3C**). Conversely, PD-L1 on tumor cells enhances glucose uptake, further depriving T cells use of this critical energy substrate (84). Collectively this body work suggests that PD-1/PD-L1 blockade can bolster T cell glycolysis to support their antitumor activity (84, 114).

CTLA-4 is a member of the immunoglobulin family on APCs and tumors that antagonizes CD28 on T cells. CTLA-4 activation on T cells suppresses their function and nutrient acquisition (115–118). Moreover, CTLA-4 downregulates the glutamine transporters (SNAT1, SNAT2) and Glut1, ultimately diminishing the bioenergetic potential of T cells in the tumor (112). LAG-3 also impaired T cell activation and proliferation (111, 119). It has been reported that LAG-3 specifically perturbs calcium influx downstream of CD3/TCR signaling, in turn preventing the differentiation of naïve T cells into effectors (120). As checkpoint blockade mediates remarkable responses in patients with a wide variety of malignancies (121, 122), it is critical to understand how IRs regulate T cell biology. Expanding our knowledge on these mechanisms will inform intelligent design of tumor immunotherapies.

T cells face many challenges to sustain effective immunity to tumors. However, it has become evident that modulating the nutritional demands of the tumor is key for sustaining proper anti-tumor T cell potential. Below we highlight the most exciting findings demonstrating how metabolically manipulating T cell *ex vivo* for adoptive immunotherapy can enhance and improve future immunotherapies.

## Modulating Metabolism to Enhance Adoptive T Cell Therapy

### Introduction to Adoptive T Cell Transfer Therapy

Some patients become resistant to checkpoint inhibition therapy. Consequently, many investigators are trying alternative therapeutic approaches that can prevent resistance or relapse, including the transfer of tumor specific T cells. Cellular therapies, such as autologous tumor infiltrating lymphocytes (TIL) or engineered chimeric antigen receptor (CAR) T cell approaches have demonstrated great potential in mediating long-lasting responses against tumors (2). Generally, whether TIL or CAR adoptive T cell therapies rely on three basic principles, a) conditioning of host with nonmyeloablative chemotherapy or total body irradiation, b) growth of T cells to large therapeutic doses and 3) treatment post-transfer with high dose IL-2 (123). This therapeutic approach holds the promise of vastly improving cancer treatment, especially for tumors rich in neoantigens, as reported for epithelial cancers such as ovarian and triple negative breast cancer (124–126). However, two major limitations for this approach are the ability to generate enough tumor specific T cells for infusion into patients and the capacity of the infused T cell products to persist long-term.

In situations where naturally arising TILs cannot be generated from a patient, gene therapy has opened the door for synthesizing T cells by directing them against tumors with chimeric antigen receptors (CAR). CD19-specific CAR T cells, designed to recognize B cell malignancies, have mediated long-lasting responses in some patients that have exhausted all other treatment options (127–129). The efficacy of these CAR T cells resulted in FDA approval of three different CD19-CAR T cell preparations thus far: two with CD28 costimulatory domains (axicabtagene ciloleucel and brexacabtagene autoleucel), and one with 4-1BB as a costimulatory domain (tisagenlecleucel). Although both TIL and CAR therapy have shown promise, sustaining prolonged and durable responses in all patients remains a challenge. Yet, the manipulation of T cells in an *ex vivo* setting provides a unique opportunity to specifically empower T cells with antitumor properties, including remodeling their metabolism, without indirect effects on the tumor. Herein we describe new advances in how TIL, CAR and TCR-based cellular therapies have been improved by altering both T cell and tumor bioenergetics.

### Metabolic Reprogramming in the Design of CAR T Cells

**Figure 4:**
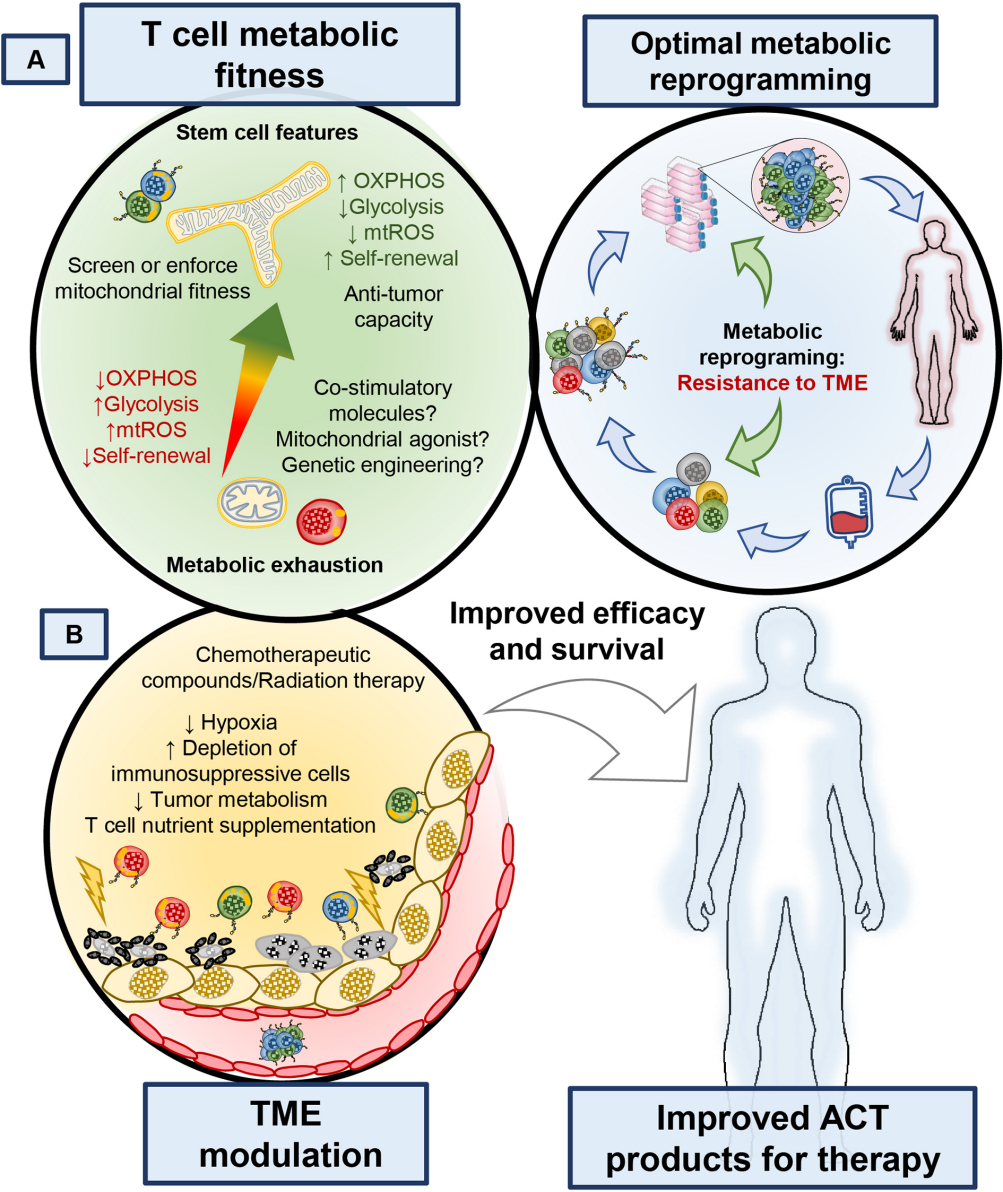
Manipulating the ability of T cells to withstand metabolic stress or altering the metabolism of tumors can enhance the therapeutic potential of T cell-based therapies. **(A)** Identifying markers that identify metabolically competent T cells, as well as understanding how small molecule compounds, biologics or receptor ligands could improve T cell metabolism will bring new targets to improve the efficacy of T cell products. **(B)** Better understanding of how the tumor microenvironment is affected by current therapies could provide new avenues to target both T cell and tumor metabolism to bolster immunotherapies. Enriching metabolically fit T cells during T cell isolation from whole blood or including metabolism modulating agents during TIL and CAR T cell expansion or altering CAR design of T cells could improve the survival of patients treated with cellular therapies.

CAR T cell construct design has evolved to include many flavors of signaling domains, kill switches, switch receptors and regulatory functions. These factors in CAR design have been reported to exquisitely control T cell functionality and selectivity against tumor targets, as reviewed previously (130). Results from early trials of CD28ζ and 4-1BBζ CAR T cells made functional differences between these cells apparent; while the CD28ζ CAR had high incidence of cytokine release syndrome and persistence on the order of months (131), the 41BBζ CAR T was able to persist on the order of years after treatment (132) and exhibited lower rates of T cell exhaustion (133). Early on, understanding of these differences was unclear; however, the June lab discovered a mechanism relating these functional differences to effects of the costimulatory domain on mitochondrial function and bioenergetics (82). 4-1BB signaling enhanced T cell bioenergetics by directly upregulating PGC1α, a transcription factor that promotes increased mitochondrial biogenesis and OXPHOS of T cells (82), supporting their long-term persistence (**Figure 4A**). In contrast, CD28ζ CAR T cells were highly glycolytic and were driven to a terminal effector phenotype (82). Further, reports have also shown that strong and chronic signaling from the CAR domain impaired T cell persistence and function due to impaired mitochondrial metabolism (133). Given these results, it is clear that the costimulatory domains incorporated into CAR T cell designs have functional and metabolic consequences which could be harnessed based on the needs of the patient. 4-1BB is part of the tumor necrosis factor related super family (TNFRSF), which consists of many other members that can be expressed in T cells such as ICOS, OX40, GITR, and CD27. Although their signaling mechanisms are known, whether they affect metabolic fitness or could empower CAR T cell persistence in patients is an active area of study.

### Cytokine Priming and Metabolic Re-Programming

The generation of TIL products is possible through the use of high dose IL-2 in tumor digests. Current protocols promote the proliferation of T cells from tumor biopsies and can yield billions of cells after weeks to months of manufacturing. However, TIL products expanded *in vitro* are fully differentiated and show features of senescence, which impairs their persistence and antitumor capacity (2, 108). In contrast, T cells generated with central or stem-cell memory properties *in vitro* have increased potential for antitumor immunity (20, 39, 134). It has long been appreciated that priming T cells with the common γ chain cytokines IL-7, IL-15 or IL-21, can generate and sustain memory T cells and have shown promise in preclinical models of adoptive T cell therapy (135). In fact, expansion of TIL from patient biopsies using a combination of the common γ chain cytokines have yielded less differentiated T cells with improved stemness features, however whether they synergize in combination to improve T cell bioenergetics remains to be fully elucidated (136, 137). Compared to IL-2 conditioning, expanding T cells with IL-15 vastly improves mitochondrial fitness, prevents overt T cell differentiation and improves tumor immunity (12, 36). Furthermore, when compared with IL-15, IL-21 appears to be most effective at preventing T cell differentiation prior to ACT and promotes greater tumor immunity than IL-15 (138); however, whether IL-21 alters T cell metabolism in a similar manner as IL-15 remains to be determined. Recently two independent reports showed that targeting IL-21 directly to T cells rather than systemic delivery in combination with PD-1 therapy improved tumor immunity. They also showed that systemic delivery of soluble IL-21 did not improve the therapeutic efficacy of PD-1 blockade, however when the anti-PD-1 antibody was fused to IL-21 synergetic improvement in tumor immunity was noticed (139, 140). There are currently many efforts to translate the use of single and combinations of these cytokines to expand TIL and CAR products for ACT, as well as novel ways to incorporate cytokine releasing switches in CAR constructs and fusion proteins. However, these studies highlight a need to better understand how and when to use these modulatory cytokines, as they may compromise T cell growth or functionality due to their effect on other immune cells within tumors.

### Inhibiting Signaling Pathways to Improve T Cell Therapies

Engagement of TCR, costimulatory molecules and cytokines mediate many internal cascades that contribute to T cell differentiation. These signals promote immunological memory; however, depending on the type and strength, these signals can also drive to T cell differentiation and exhaustion (141). An attractive approach is to use pharmacologic inhibitors against signaling cascades downstream of these signaling cues (**Figure 4A**) to generate antitumor T cells with durable memory traits. Canonical signaling downstream of T cell activation turns on the PI3K/ATK/mTOR signaling pathway and leads to T cell effector differentiation (142). This signaling axis is critical for rewiring metabolism to enable growth, protein translation and function in all proliferating cells, thus using compounds that target components of this pathway is a sensible approach to modulate T cell biology to improve their anti-tumor potential. Our lab and others have shown that targeting the delta subunit of PI3K, which is expressed specifically in lymphocytes, generates T cells with a less differentiated state (143), including murine and human antitumor CD8^+^ CTLs or CD4^+^ Th17 cells (144, 145). In fact, Dwyer et al. reported that blocking the PI3Kδ or PI3Kγ subunits were most advantageous for the production of highly effective anti-tumor T cells compared to those treated with drugs that inhibited both PI3Kδ and PI3Kγ subunits (146). Although is predicted that PI3K inhibition should dampen glucose metabolism and reciprocally improve T cell mitochondrial function it is still to be explored if selectively inhibiting the delta or gamma subunit have lasting effects on T cell metabolism reprograming or mitochondrial fitness (146). Downstream of PI3K, Akt blockade was also found to increase T cell stemness as well as FAO metabolism without perturbation of glycolysis upon restimulation (147, 148). Furthermore, inhibition of mTOR with rapamycin generates T cells that resemble a rare stem-memory like T cell population with enhanced survival capacity, mitochondrial respiration and lasting persistence in hosts (149–151). These findings reveal an interesting approach in modulating T cell differentiation and metabolism, which endow T cells with enhanced tumor-killing capacity. However, the question is raised as to which approach is most effective and what mechanisms govern the efficacy of this therapeutic inhibition during ACT expansion. A potential mechanism is that inhibiting the PI3K/Akt/mTOR pathway leads to enhanced autophagy, which is a well described homeostatic process involved promoting T cell memory and mitochondrial fitness (152, 153). This idea is further strengthened by a new report shown an important role for T cell intrinsic mitochondrial regulation by autophagy as an important part sustained immunity against tumors (154).

Another clue as to how blocking signaling cascades may overlap to improve T cell therapies was recently identified by the Restifo group (155). They used a multi-phenotype CRISPR screen to identify more than 25 targets downstream of T cell activation. They identified the stress response p38 MAP kinase as a key driver involved in preventing T cell mediated tumor immunity (155). This finding reinforces previous studies that elegantly demonstrated that ER stress, a target regulated by p38, impairs intratumoral T cell protein translation of cytotoxic molecules and regulates mitochondrial and T cell exhaustion (156–159). Nonetheless, the current efforts exploring inhibition of these key signaling pathways *in vitro* may provide TIL and CAR T cell with enhanced bioenergetics, persistence and anti-tumor capacity during their expansion. A potential benefit of using inhibitors of these key proliferation pathways is their effect on tumor and other suppressive immune cells, such as T_regs_ (160, 161) and myeloid cells (162) as they are sensitive to PI3K inhibition and may enhance the expansion of TIL. Based on current the literature, inhibition of growth and differentiation pathways such as the PI3K/AKT/mTOR signaling axis directly alters the development and metabolic programing of T cells *in vitro*, which improves their bioenergetics and persistence *in vivo* (**Figure 4A**).

### Exploiting Nutritional Stress *in Vitro* for Beneficial Metabolic Adaptations

Recent reports show that T cells expanded in the presence of metabolic stress are surprisingly better at delaying tumor growth (103, 163). Although this finding is counterintuitive, this discovery may be explained by the ability of T cells to adapt to scarce environments by upregulating alternative sources of fuel through metabolic adaptations. For example, Sukumar et al. found that depriving T cells of glucose *in vitro* increased the number of less differentiated CTLs and supported their stem and central memory profile. These glucose-starved T cells regained potent effector functions in the tumor when infused into mice (163). Moreover, these cells upregulated AMPK activity, known to enhance mitochondrial respiration and fatty acid usage, and mediated robust regression of melanoma compared to conventionally cultured T cells (163). Most recently this finding has been supported by transient glucose restriction which improves T cell immunity against tumors *via* increased pentose phosphate pathway activity (164). Note that this study is not diminishing the importance of glucose for T cell survival and effector functions. Instead, it highlights the unexpected finding that the biology of T cells can be altered simply by transiently denying them this metabolite *in vitro*, an adaptation likely to be driven by mitochondrial compensation (62). In fact, T cells engineered to overexpress the gluconeogenesis enzyme phosphoenolpyruvate carboxykinase (PCK) can improve antitumor immunity, putatively by increasing the pool of glucose available to enter glycolysis and other ancillary pathways like the pentose phosphate pathway (165).

Glucose availability is a requirement for T cell mediated immunity *in vivo*, so how does depriving T cells of glucose *in vitro* enhance tumor immunity? An explanation could be provided by recent work showing that nutritional deprivation can be a double-edged sword depending on the context. In this work, priming T cells *in vitro* with high potassium concentrations lead to metabolic reprogramming to increase Acetyl Co-A Synthase (ACCS1) which enhanced mitochondrial respiration, conferred stem memory qualities in T cell and enhanced tumor immunity *in vivo (*49, 153). This data revealed that priming T cells *in vitro* with nutritional deprivation can improve antitumor activity, in part due to improved bioenergetic plasticity in a similar fashion as depriving glucose *in vitro*. Pressuring T cells to undergo metabolic adaptations that allow survival under cellular stress, such as promoting mitochondrial biogenesis, enhancing mitochondrial respiration, or enhancing ancillary pathways such as PPP or gluconeogenesis can benefit tumor control.

### Nutritional Support for Anti-tumor T Cells

Collectively, this rich body of work on T cell metabolism highlights the need for T cells to adapt and use alternative fuel sources to thrive in the harsh tumor microenvironment. So, the question is posed, which fuels are most effective at supporting T cell antitumor activity? Recent work suggests that select amino acids and nucleotides may contribute. For example, supplementation of L-arginine *in vitro* and *in vivo* improves T cell tumoricidal activity by enhancing their memory formation and mitochondrial respiration (166). Additionally, supplementing inosine (a nucleoside capable of entering the central carbon pathways of glycolysis and the pentose phosphate pathway) under glucose deprivation enhanced the ability of T cells to clear tumors in mice (59). Identifying unique metabolites to augment cancer immunotherapy is attractive, as they can be delivered directly into T cell cultures or *in vivo* without overt expected side effects. A recent report suggests that highly therapeutic CD26^high^ T cells might have those properties (167), as CD26 docks adenosine deaminase (ADA), which cleaves suppressive adenosine [produced by tumors and T_regs_ (105)] into inosine an important precursor for nucleotide synthesis and feedback into one carbon metabolism (59). This idea is particularly attractive given how potent CD26^high^ T cells are at ablating large tumors, and suggest that ADA-induced inosine might play a role in their potency. Furthermore, methionine is a metabolite that enters the central carbon cycle and is a required amino acid for supporting T cells with effector properties (60, 61). *In vivo*, supplementing T cells with surplus L-arginine, inosine or potentially methionine could be an attractive way to enhance tumor immunity by providing alternative fuels for T cells exogenously (**Figure 4B**).

### Direct Mitochondrial Agonists to Enhance Anti-tumor T Cells

Modulating nutrients to directly fuel T cells within tumors may not be the most efficient way to help their support their bioenergetic needs. Instead, directly stimulating the mitochondrial function of T cells using pharmacologic agonists might be more effective (**Figure 4B**). In fact, several reports have shown that small molecule agonists of AMPK, mTOR and PPARα or γ in combination with immune checkpoint blockade (ICB) therapy can promote mitochondrial function in T cells, leading to a positive immune response against tumors (168). For example, PD-L1 blockade in combination with an agonist of peroxisome proliferator–activated receptor γ and co-activator of PGC1α was remarkably effective at mediating curative responses in mice with melanoma (169). This approach is supported by a recent study revealing that T cell mitochondrial function is a marker for responsiveness in patients treated with ICB (168). Thus, it is likely that mitochondrial T cell health and regulation may play an important role in patient responses to immunotherapy (62, 68). Also, survival cues (such as AMPK, PKA and the Sirtuin family of acetylating enzymes) regulate T cell metabolism under energetic stress. This action improves T cell survival *via* bolstering mitochondrial biogenesis (PGC1a), remodeling (fused mitochondria) and recycling (i.e. mitochondrial autophagy) (70, 81, 154). However, direct perturbation of the tumor itself is also likely to modulate metabolites that promote T cell immunity (**Figure 4B**). Many investigators are thus focused on combining radiation or chemotherapy with ICB to help patients, but exactly how these therapies impact the nutrient tug of war between tumor and T cells is still up for debate as well as whether these approaches can be exploited for the expansion of TIL and CAR T cells.

## Manipulation of Tumor Cell Metabolism

New insights into the metabolic requirements for tumors has sparked interest in manipulating their metabolism to improve immunity (54, 102). Nonetheless, there is limited but promising data regarding the benefit of combining current chemotherapeutic strategies or inhibition of tumor metabolism. Although glucose availability is a key determinant of T cell response, the heterogenous nature of the tumor warrants exploration of multiple targets (55, 57).

Preventing tumors from using the nutrients they need to survive can provide an advantage for T cell effector functions. For example, targeting the lactate dehydrogenase (LDHA), an enzyme that converts pyruvate to lactate and regeneration of NAD+ in tumors, improves T and NK cell function (47, 170, 171). Additionally, altering the hypoxic tumor environment can improve the therapeutic potential of ICB and adoptive transfer therapies, given the critical role of HIF1-α in altering the metabolic requirements of tumors under oxygen stress (172). For example, metformin plus PD-1 therapy enhanced the antitumor capacity of endogenous T cells in mice, in part by reducing the hypoxic nature of the melanoma (173). Another approach to target the tumor to augment immunity has been the neutralization of the highly acidic TME with sodium bicarbonate or other proton pump inhibitors prior to ICB or ACT (174). Combination of tumor metabolism inhibition and chemotherapeutic regiments may relieve the nutritional tug of war between tumors and T cells (93, 175). One promising strategy is to block glutamine metabolism within tumors, as this can also empower T cell immunity, a remarkable feat for single chemotherapy agents (58). Finally, another example of targeting the tumor to augment outcomes is found in pre-conditioning patients with systemic cisplatin to enhance T cell immunity at a secondary tumor site following radiotherapy, also known as the abscopal effect (176, 177). Thus, identifying FDA approved chemotherapeutics that alter tumor metabolism to augment the therapeutic potential of immunotherapies will be key to improve current therapeutic approaches (**Figure 4**).

## Summary and Perspectives

It has 70 years since the first studies on T cell metabolism (178). The importance of T cell bioenergetics and its effect on immunity are gaining a new level of appreciation today and are being explored by multiple investigators. Yet, many key questions remain unanswered about how T cell metabolism impacts immunotherapy. For example, how do expression of inhibitory receptors and co-stimulatory molecules [such as LAG-3, TIM3, ICOS and other TNFRSF receptors (i.e. OX40, 4-1BB] impact T cell and tumor metabolism? Moreover, how do “suffering” T cells preconditioned under nutritional deficits gain antitumor activity *in vivo*? Insights into these mechanisms will be critical to design optimal therapies as mono- or combination approaches.

Herein, we have highlighted the myriad of ways metabolism is emerging as a major target for next generation immunotherapies. While the optimal therapeutic approach is unclear, promising strategies include targeting the tumor/immune axis either altogether or as individual branches. Chemotherapy and irradiation as preconditioning agents hinder the tumor directly, permit release of antigens and host immune activation. After effective tumor priming, administration of potent immune activating agents can help overcome immune evasion by the tumor. These immune therapies include checkpoint inhibitors, costimulatory agonists, and adoptively transferred T cells, each have the potential to harness a metabolic advantage for antitumor immune cells. Additionally, direct administration of agents which alter nutrient plasticity or promote metabolic adaptation of T cells over tumors could also synergize. However, as highlighted by the effect of tumor glycolysis and CTLA-4 blockade and the ability of other immunosuppressive cells to benefit from metabolism modulators there is always a possibility that fostering a metabolic advantage for the T cells in the tumor could also benefit the immunosuppressive microenvironment of the tumor, thus defining the timing and sequence of intervention is a challenge that needs to be addressed. One advantage of adoptive T cell therapy as an alternative, is the flexibility to manipulate the T cell directly obviating the challenge of competing immunosuppressive cells and the tumor. However, there are challenges that remain in designing optimal methods to reliably and potently alter the metabolism and function of *ex vivo* expanded T cells. This body of work suggest that by using inhibitors of key differentiation/stress pathways or conditioning with cytokines or co-receptors that improve metabolic function, we could provide the competitive advantage needed to ablate tumors long-term in patients. Regardless of the specific method, metabolic rewiring is likely to play a significant role in eliciting durable and long-lasting immunity in tumors resistant to conventional therapies.

## Author Contributions

GR and CP designed, wrote and edited the manuscript. HK, CD, AS, MW, AR and JT provided feedback and edited the manuscript. All authors contributed to the article and approved the submitted version.

## Funding

This work was supported by the NIH Training grant T32 5T32GM008716-19 and MUSC CGS Provost Scholarship to GR. NIH Training grant T32 GM08716 and NCI F30 243307 to HK. NIH training grant T32 AI132164-01 to CD. Hollings Cancer Center Graduate Fellowship to AS. NIH R50 CA233186 to MW. NIH R01 CA175061 and R01 CA208514 grants, KL2 South Carolina Clinical and Translational Research grant UL1 TR000062, ACS-IRG grant 016623-004 and MUSC Start-up funds to CP.

## Conflict of Interest

The authors declare that the research was conducted in the absence of any commercial or financial relationships that could be construed as a potential conflict of interest.
